# Excision of the Ovaries

**Published:** 1899-03

**Authors:** Mary I. Hall Williams

**Affiliations:** Boston; The Nook, Penzance, Cornwall, England


### EXCISION OF THE OVARIES.

THE NOOK, PENZANCE, CORNWALL, ENGLAND,

JANUARY 26th, 1899.

EDITOR OF THE HOMŒOPATHIC PHYSICIAN:—On page 433 of THE HOMŒOPATHIC PHYSICIAN for October there is a paragraph deprecating justly the excision of the ovary, especially for the purpose of the avoidance of child-bearing.

May it not be considered that when child-bearing is or becomes distasteful to a woman it also becomes unstatesmanlike to enforce it; because of the way such children must be influenced, and of the kind of men and women to be thus supplied to the State? Does it not take us further than the poor woman and her surgeon, and turn us backward to the husband and the law?

I am faithfully yours,

MARY I. HALL WILLIAMS, M. D., Boston.

